# Blood Management and Risk Assessment for Transfusion in Pediatric Spinal Deformity Surgery

**DOI:** 10.1155/2020/8246309

**Published:** 2020-05-07

**Authors:** Pedro Fernandes, Joaquim Soares do Brito, Isabel Flores, Jacinto Monteiro

**Affiliations:** ^1^Orthopedic Department, University Hospital of Santa Maria, Lisbon, Portugal; ^2^ISCTE, IF Data, Lisbon, Portugal

## Abstract

**Objectives:**

Evaluate the impact of a Quality and Safety Program (QSP) on the reduction of blood loss and transfusion needs in pediatric spinal deformity surgery, while defining risk factors for transfusion.

**Background:**

Multimodal plan aiming to minimize transfusion needs has been shown to reduce transfusions and index rates in spinal deformity surgery. Anticipating blood loss and transfusion may help direct resources to patient needs or encourage reconsideration of the surgical plan.

**Methods:**

This is a single-center retrospective study of prospectively collected data. Impact of this multimodal plan was studied on idiopathic deformities (Group A, 109 patients) and scoliosis associated with syndromic, neuromuscular, and muscular dystrophies (Group B, 100 patients), both before and after QSP.

**Results:**

A decrease in total estimated blood loss was observed. In Group A, transfused patients decreased from 83.7% to 28% (*p* < 0.001, odds: 0.077), and, in Group B, from 98.7% to 66% (*p* < 0.01, odds: 0.038). Pearson's correlation identified patient body weight (*r* = 0.245, *p*=0.001) and Cobb angle (*r* = 0.175, *p*=0.017) as factors related to blood loss. A linear regression model to estimate hematic losses revealed that only body weight and transfusion showed predictive power, resulting in a low predictive model (*R*^2^ = 0.156; *F*(3,167) = 15.483, *p* < 0.001). A mediated model to explain blood loss was built based on a set of variables influencing transfusion which is, in turn, related to blood loss.

**Conclusion:**

Transfusion needs in scoliosis surgery can be substantially reduced following a multimodal approach. The success of a program is strongly dependent on team effort, and the introduction of a risk assessment tool for transfusion needs indirectly assesses surgical risk, thus allowing relocation of resources to decrease blood loss.

## 1. Introduction

Scoliosis surgery is considered one of the most complex and invasive surgical procedures in pediatric orthopedics. Despite significant bleeding due to exposure length, muscle dissection, and vertebral instrumentation, a clear definition of massive bleeding is still lacking. In fact, hemorrhage during scoliosis surgery can be major, with some authors reporting blood losses up to 4.5 liters per surgery [1, 2]. And if the consequences of hematic losses are not clear enough, the fact remains that these procedures frequently lead to blood plasma coagulation factor and platelet transfusions [2]. Therefore, the risk of viral infection, adverse events, alloimmunization, and bacterial sepsis are all well known after transfusion of blood derivatives [3, 4]. More recently, several authors have correlated the transfusion of allogenic blood with postoperative surgical site infection [4–6].

Several measures can be implemented to prevent excessive blood loss during surgery, such as correct positioning of the patient on the operative table, the use of hypotensive anesthesia during spinal exposure, and hemodilution to reduce initial blood loss. The use of a cell-salvage system, tranexamic acid, and an adequate surgical technique which also applies local hemostatics represents some of the other measures which may minimize blood loss [7–10]. At the same time, a restrictive transfusion policy will help to minimize the transfusion rate. In 2011, the authors introduced a Quality and Safety Program (QSP) for pediatric spinal deformity surgery aiming, primarily, to decrease the rate of postoperative surgical site infection. This also included a multimodal approach to reduce transfusion needs (MAPTn). In this study, the authors present the impact of the measures implemented to reduce the transfusion rate and transfusion index, thus identifying several risk factors for relevant blood loss.

## 2. Materials and Methods

The study is an outcome comparative analysis between two pediatric populations submitted to posterior spinal fusion for scoliosis. Patients were divided by etiology and whether they were exposed or not to a multimodal approach to decrease transfusion needs. This is a retrospective study with prospectively collected data. All patients were operated on by the lead author with the same exposure and dissection technique after two years of practice following a one-year spine fellowship.

### 2.1. Inclusion Criteria

Patients under 21 years of age diagnosed with scoliosis and kyphosis who underwent surgery in our institution between 2006 and 2016 were included. All deformities requiring posterior instrumentation of five or more levels, regardless of previous anterior or posterior release, were considered as inclusion criteria. Traumatic deformities and those caused by spondylodiscitis were not included.

### 2.2. Sample

The study included 209 patients with a minimum follow-up of 24 months. Patients with idiopathic deformities (scoliosis and Scheuermann's kyphosis) and congenital deformities treated as idiopathic were included in Group A (109 patients), whereas patients with diagnoses of neuromuscular and syndromic scoliosis and muscular dystrophy formed Group B (100 patients). Considering exposure to QSP, each group was then divided into two subgroups: subgroup A1 (49 patients) and subgroup B1 (51 patients) comprised the patients who underwent surgery between January 2006 and June 2011; subgroup A2 (60 patients) and subgroup B2 (49 patients) encompassed the patients operated between July 2011 and July 2016, after QSP implementation. The year 2011 was divided, as we began to implement the measures in the second trimester, and implementation was considered to be fully completed by July of the same year.

### 2.3. Multimodal Program to Prevent Blood Loss and Transfusion

#### 2.3.1. Preoperative Period

Patients were evaluated according to their comorbidities, and hemoglobin was raised above 12 gr/dl with iron and erythropoietin when needed. Autologous blood donation was suspended in idiopathic deformities as all patients presented an important decrease in preoperative hemoglobin. In the case of neuromuscular patients, a multidisciplinary approach was introduced in the treatment decision, including a preoperative conference with neurology and pneumology specialists and physiatrists. With these measures, the ultimate goal was to detect and correct any undiagnosed anemia by improving the preoperative hemoglobin level and, when possible, addressing the nutritional status.

#### 2.3.2. Intraoperative Period

New intraoperative measures involved the introduction of tranexamic acid in an initial dosage varying between 10 and 30 mg/Kg, followed by a perfusion of 3 to 5 mg/kg/h. Remifentanil infusions progressively replaced the use of fentanyl, in order to allow a better control of hypotension mainly during exposure, where a mean arterial pressure (MAP) of 65 mm Hg was the goal. In both periods, the same routine was kept during incision with skin infiltration with 1 mg of epinephrine/500 ml of saline solution, as well as a meticulous surgical technique aiming to decrease bleeding throughout the procedure. Fascia was closed water tight with drains over it.

#### 2.3.3. Postoperative Period

A restrictive transfusion index was initiated, thus avoiding transfusion with hemoglobin above 7 gr/dl or hematocrit (HCT) > 21, following an uneventful surgery and no clinical signs of hemodynamical instability. Concurrently, autotransfusion, a practice often used in idiopathic scoliosis surgery during the pre-QSP period, was abandoned, and, when available, a cell saver was introduced.

#### 2.3.4. Program Monitoring

Until the end of 2012, colleagues and staff were persuaded to deviate from usual practice by a unit protocol, supervised by the lead surgeon, and later, the multimodal approach for decreasing transfusion needs started to be more general imperative, with the participation of the Anesthesia and the Hemotherapy Departments.

#### 2.3.5. Outcomes

Estimated blood loss (EBL), blood loss per blood volume (weight multiplied by 0.70), and blood loss per instrumented levels were considered as relevant outcomes. Hemoglobin and hematocrit levels were measured at four different times (preoperative, intraoperative, postoperative first day, and lowest level during admission). Finally, transfusion rate and number of transfused units per patient were compared.

### 2.4. Statistical Analysis

Data were registered in a Microsoft Excel spreadsheet, and statistical analysis was performed with IBM SPSS Statistics 23 software. All continuous variables were tested for the normality of their distributions: when normality was met, linear models were used and data were represented in mean ± standard deviation. D-Cohen was calculated for effect size evaluation, being considered as relevant when above 0.2. In the absence of normality, the Mann–Whitney *U*-test was applied with data represented by the median ± interquartile interval and effect size was calculated by eta-squared for continuous variables. Percentages were compared by applying a Pearson chi-squared test with effect size given by odds ratio (OR) adjusted to postprogram. The gathered data were applied to perform linear and logistic regression models, and Pearson's correlations were used to assess the risk factors for blood loss. Multivariate analysis was performed to find a model that could estimate blood loss. The model found turned out to be a mediated model, with a set of variables influencing transfusion probability, which, in turn, had an impact on blood loss volume. This model made use of multiple logistic regression to predict transfusion (0 = no; 1 = yes) and a multiple linear regression model to predict blood loss volume (continuous variable). Using predicted probabilities resulting from the logistic regression, sensitivity and sensibility were calculated and anchored on a ROC curve, The analyzed variables were weight; preoperative Hb; Cobb major; tranexamic acid (0 = no; 1 = yes); anesthetic (0: fentanyl; 1-remifentanil); diagnosis (*A* = 0: idiopathic, 1: Scheuermann's, and 2: congenital) (*B* = 3: neuromuscular, 4: syndromic, and 5: dystrophy).

## 3. Results

In Tables 1 and 2, we represent the demographic characteristics, deformity magnitude, instrumented levels, and length of surgery for the populations under study before and after the implementation of MAPTn. In Group A, despite the significances obtained in the first analysis, size effect, tested with odds ratio (OR) adjusted to postprogram and eta-squared, only showed the difference in gender as relevant, with more female patients in the postprogram period (*p* < 0.05; OR 2.9: 95% CI [1.18–7.09]). Before the introduction of the MAPTn, the median hemoglobin was 12.5 ± 1.8, while at postprogram, it was 13.8 ± 1.3 (*p*=0.01, effect dimension: 0.11). One or two units of autologous blood were collected from 20 patients during the pre-QSP period. At post-QSP, one patient had anemia (<12 gr/ml) corrected with erythropoietin (Jehovah's witness), while eight others had their low levels of hemoglobin improved with iron (one intravenously and seven orally).

Deformity severity was equal in both periods, and no relevant differences were considered in the number of levels fused (*p*=0.01, ED = 0.05) and operative time (*p* < 0.001, ED = 0.077) in the post-QSP period. In Group B, a relevant difference was registered only for anterior releases, more often performed in the preprogram period (*p* < 0.05; OR 0.352; [CI −0.12–1.0]).

Estimated blood losses (EBL), blood loss per blood volume (EBLV) and per instrumented level (EBLL), were compared in both populations between preprogram and postprogram periods. It should be noted that a decline was observed in blood loss, both in idiopathic deformities and deformities associated with other etiologies (Tables 3 and 4). Although differences were not statistically significant, a 40 cc median reduction in EBL was observed at sample level in idiopathic deformities and a 100 cc median reduction in syndromic and neuromuscular scoliosis, representing a 8% and a 16% drop in hematic losses, respectively. Cell saver was used in only seven patients during surgery after MAPTn introduction (6 in group A and 1 in group B).

As previously stated, in Group A, the statistical analysis only revealed a trend towards higher preoperative hemoglobin at postprogram, even though its effect dimension was low. Intraoperative, postoperative day 1, and lowest levels of hemoglobin (Hg) and hematocrit (Htc) in the perioperative period, before and after MAPTn, were similar for both groups (Table 2). On the other hand, the transfusion rate and the number of units transfused per patient were markedly reduced with no significant difference, as referred in the Hg/Htc profile. In Group A, the number of transfused patients declined from 83.7% to 28% (*p* < 0.001; OR 0.077: 95% CI [0.03–0.198]), and the number of units transfused per patient decreased from 2 ± 1 to 0 ± 1 (*p* < 0.001; ED = 037). A less impressive but relevant difference was observed in Group B (neuromuscular and syndromic scoliosis and dystrophies), where the transfusion rate fell from 98.7% to 66% (*p* < 0.001; OR 0.038: 95% CI (0.05–0.30) and the number of units transfused fell from two units to less than one per patient (*p* < 0.01; DE = 0.29) (Table 4).

In order to evaluate the effect of our program in blood loss, a multiple linear regression model was performed introducing confounding factors. The predictors were patient age and weight, fused levels, and operative time. As can be seen in Table 5, the model, with a low to moderate predictability level (*R*^2^ = 0.209), could predict a decrease in blood loss in Groups A and B following the adoption of MAPTn (beta = −0.433; *p* = 0.003). Even though, if at sample level, diagnostic B shows a higher correlation with blood loss, it is not significant for EBL and EBL/level and therefore cannot be extrapolated to other samples/populations. This relation was only relevant for EBL/volemia (beta = 0.539; *p* = 0.001). Surgery duration (beta = 0.302; *p* < 0.001) and age (beta = 0.163; *p* = 0.034) were both responsible for higher EBL blood losses.

After excluding patients with incomplete data, independent correlations of different relevant factors with the estimated hematic losses were determined for 155 patients. In this analysis, only weight and Major Cobb deformity angle (CobbM) proved to be significant, even though Pearson's correlation values were low (weight: *r* = 0.245, *p*=0.001; CobbM: *r* = 0.175, *p*=0.017). In a linear regression model to quantitatively estimate hematic losses, only weight and transfusion (transfused = 1, not transfused = 0) were relevant but presented a low predictive capacity (*R*^2^ = 0.156; *F* (3,167) = 15.483, *p* < 0.001).

**Figure 1:**
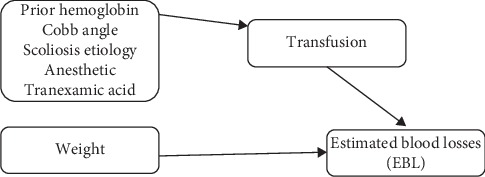
Process model mediated by transfusion in the calculation of transfusion probability.

Even though this was our predictive model for final estimated blood loss (EBL), we believe that the effects of the remaining variables could play a role, not in EBL, but in the prediction of transfusion. Therefore, a new process model mediated by transfusion was built. First, the transfusion probability was calculated, and then, its association with EBL was established (Figure 1). All the continuous variables were standardized in order to have comparable relative effects (beta) between continuous and categorical dummy variables. The result was reasonable to the high explanatory power model for the possibility of transfusion with pseudo *R*^2^ = 0.559. Transfusion probability was calculated with a logistic regression where all the variables except weight (which directly influences EBL) were introduced as transfusion predictive variables. In this model, redundant variables were the ones which indirectly influenced EBL, since they played a decisive role in transfusion probability (Table 6).

The higher the previous Hb was, the lower the probability of transfusion became, as well as the need to use tranexamic acid (Trax = 1) and remifentanil (Anest = 1). On the other hand, the transfusion probability increased with a higher Cobb Major angle and diagnosis B (pathologies 3, 4, and 5 coded as 1), namely, cerebral palsy, syndromic scoliosis, and muscular dystrophy.

**Figure 2:**
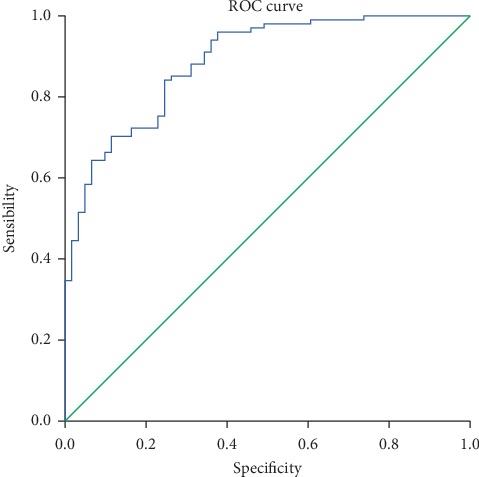
ROC curve for the explanatory model for transfusion calculated according to the estimated probabilities for the event.

A complementary use for logistic regression was to estimate the risk of transfusion in each patient. A ROC curve depicting transfusion probability, a variable calculated from the previous logistic regression, revealed that the calculated probability presented a good diagnostic power, considering the area under the ROC curve = 0.887 (95% CI: 0.838 and 0.937; *p* < 0.001) (Figure 2).

This ROC curve appeared to have three selectable points, depending on whether our gains would be greater with sensitivity (fewer false negatives) or specificity (fewer false positives), or conversely, depending on whether we would only intend to maximize balance between both. Even though the cutting point where greater balance was achieved was 0.632 (sensitivity 0.842 and specificity 0.75), in order to obtain greater sensitivity (96%), the selected cutting point was 0.29, since it maximized the global percentage to 83.3%, according to the diagnostic table.

## 4. Discussion

Hematic losses in spinal surgery are one of the main problems that spine surgeons face in the treatment of spinal deformities. These problems are not only due to the magnitude of the deformities but also due to the variability and unpredictability which characterize them, since they are based on multiple factors. Thus, transfusions are frequent, and several studies correlate them with an increase in the risk of surgical site infection, late wound healing, longer hospital stay, and death [11, 12]. According to Shapiro et al., hemorrhage depends on the type of scoliosis, approach, operative time, and length of procedure, making blood volume loss almost impossible to predict [13]. In this study, neuromuscular scoliosis presents the greatest risk of blood loss and, among this category of deformities, Duchene muscular dystrophy is the most problematic [13]. Our study confirms these observations of greater blood loss in the neuromuscular scoliosis group, mainly when analyzed in terms of blood volume and instrumented levels, where the difference was impressive.

When analyzing the perioperative hemoglobin profiles, we verified that, in Group A, patients presented higher hemoglobin levels in the post-QSP period, which may have corresponded to better patient prehabilitation in idiopathic curves, to a certain extent by prior anemia correction, but most likely due to the suspension of the autotransfusion program. The advantages of autotransfusion have recently been questioned, given the considerable costs and the increase in the transfusion index [14–16]. During the pre-QSP period, we often observed transfusions with hematocrit above 30% and hemoglobin of 10 gr/dl related with the more liberal transfusion policy followed by the anesthesiologist and colleagues in the pediatric intensive care unit, especially if patients received their own blood units, a policy, as we now know, not completely innocuous, considering the rate of adverse reactions associated with autotransfusions [16].

According to several series, the transfusion rate in spinal surgery has been considerable, varying between 36% and 75% [1, 2, 10, 17–19]. Actually, these were our rates before the implementation of the multimodal approach to decrease transfusion needs which was responsible for the marked drop in the transfusion rate in our patients. While before the program, the transfusion rate varied from 83.7% to 98.7%, after the program, a variance between 28.3% and 66% was registered, being highly dependent on the etiology of the scoliosis.

MacShane et al. published their results regarding the reduction of transfusions of allogeneic products based on a program focused on the increase of prior hemoglobin and the avoidance of autotransfusion. With these two measures, the authors were able to reduce the number of transfused patients from 63% to 14% [19]. Hassan et al. were also able to decrease the transfusion rate in idiopathic scoliosis in 36% to 1.7% of the cases [10]. In spite of emphasizing the cumulative effect of the various measures implemented, the authors believed that the use of antifibrinolytics such as tranexamic acid seemed to be a key factor in reducing intraoperative blood loss [20–23]. In 2008, a Cochrane review based on six studies compared antifibrinolytics with placebo and revealed that these pharmaceuticals reduced an average of 327 ml in the blood volume transfused with no additional morbidity, as well as an average of 427 ml in total blood losses [24]. Considering the selected publications and despite all the gathered data, the number of children who underwent scoliosis surgery and no longer required transfusion was not clear [24]. McLeod et al. published an article where the use of tranexamic acid in pediatric scoliosis surgery in several medical centers of the United States was analyzed [20]. According to their results, away from clinical trial scenarios and with a universe of 2,722 idiopathic scoliosis and 1,547 neuromuscular scoliosis, only aminocaproic acid was capable of reducing the number of transfusions in idiopathic scoliosis. However, in the neuromuscular scoliosis group, none of the antifibrinolytics, including tranexamic acid, decreased the odds ratio for transfusions. The transfusion rate was of 24% for idiopathic scoliosis and 43% for neuromuscular scoliosis. The explanation may well lie in the different dose regimes that can vary substantially between centers [22, 23, 25, 26]. Johnson et al., when comparing a 50 mg/Kg *bolus* regimen followed by a maintenance dose of 5 mg/kg/h with a low-dose regimen of 10 mg/kg followed by 1 mg/Kg/h, showed a clear reduction in losses and transfusions in the highest-dose group [26]. Nevertheless, concerns have been essentially focused on the safety profile by considering the thrombogenic effect of antifibrinolytics although several authors do attest for the safety of the pharmaceutical regarding thrombosis, pulmonary thromboembolism, or hemodynamic instability [27, 28]. In a study comparing 32 patients who were administered a high dose of tranexamic acid (100 mg/kg bolus and 10 mg/kg/h perfusion) with 32 patients who received a low dose of the same agent (10 mg/kg bolus and 1 mg/kg/h perfusion), Xie et al. did not find any significant difference in complications between both regimes [29]. According to the authors, no deep venous thrombosis, thromboembolism, kidney failure, seizures, or myocardial infarction were observed. Moreover, a high-dose regimen was clearly demonstrated to reduce blood losses and transfusions in teenager idiopathic scoliosis surgery, unlike the low-dose regimen, where no relevant differences were observed in the outcomes [29].

In our sample, since we had different dose regimes for tranexamic acid administration which were highly dependent on the anesthetist and depicted their concerns regarding the safety profile of this drug, it is possible to infer that the success of our QSP in reducing blood losses and transfusions overcame this factor. Concurrently to tranexamic acid introduction, a progressive preference for remifentanil, an easy and safe drug to use in pediatrics, improved the maintenance of controlled hypotension during surgery [30, 31]. Finally, the restrictive policy may have been one of the most important measures to decrease transfusion rates. From our experience, this measure may have been one of the most difficult to implement, as it took significant time for our colleagues to adhere to it. Transfusion restrictive policies have been implemented for several years, with a well-demonstrated safety profile even in an adult population admitted to intensive care units [32]. In pediatrics, significant variability in transfusion policies can be found, even if it is known that hemodynamically stable children with no active hemorrhage tolerate a decrease in oxygen tension arising from moderate anemia relatively well [33, 34]. Lacroix et al. showed that a restrictive transfusion policy with a threshold below 7 gr/dL applied to children hospitalized in intensive care units significantly reduced transfusions with no increased morbidity or mortality [35].

Thompson et al. tried to identify factors which could be correlated with massive hematic losses [36]. The number of instrumented levels was the variable with the greatest impact, especially in surgeries above 12 instrumented levels. Yu et al. correlated low weight, Cobb angles above 50°, more than 6 instrumented levels, and the introduction of osteotomy with the massive loss (>30% of blood volume) that occurred in 59.9% of their patients [37]. A predictive model for transfusion was developed by Vitale et al. based on multivariate analysis and logistic regression [38]. Determinant factors were diagnosis (odds: 2.02), curve degree (odds: 1.012), and use of erythropoietin (odds: 0.29) [38]. In a different model and adult surgery, Lenoir et al. considered age over 50 years, low preoperative hemoglobin, fusion of more than two levels, and the use of transpedicular osteotomy as predictive factors for transfusion [39]. Based on our data, prior hemoglobin, diagnosis of scoliosis, Cobb angle, and two anesthetic factors, the use of tranexamic acid and of remifentanil, were correlated with transfusion. The model we developed, which introduces patient's and anesthetic data in the equation, presents an 83% discriminatory value, being therefore a robust model for the determination of the probability of transfusion. It not only possesses the particular feature of integrating data from the patient and the surgery, but it also gives the anesthetist an equally important role in the probability of transfusion, depending on the use of an antifibrinolytic and the selection of the opioid during anesthesia, thus emphasizing the importance of team work. The information generated by these models provide us with an idea of the surgical risk and may also help concentrate resources for adequate optimization of the clinical condition of the patient, maximize loss-saving strategies, or, if required, reevaluate the aggressiveness of the surgery.

## Figures and Tables

**Table 1:** Characterization of the population under study before and after the implementation of the QSP program (Group A).

Characteristic	Before QSP (A1) % (N)	After QSP (A2) % (N)	Pearson chi-squared (Mann–Whitney)	*p* value	OR (95% CI) (eta-squared)
Gender	63% (49)	83.3% (60)	5.69	0.017	2.9 [1.18; 7.09]
Weight (kg)	57 ± 19.5 (36)	50 ± 12 (56)	−3.11	0.002	0.1
Age (years)	15 ± 3(49)	14 ± 2 (60)	0.219	0.820	0.0004
Cobb angle (°)	64 ± 25.75 (49)	62.3 ± 24.7 (60)	−0.198	0.843	0.0003
Adit. procedure^*∗*^	22% (11)	10% (6)	3.175	0.076	0.384 [0.13; 1.13]
Pre-op hb	12.5 ± 1.8 (49)	13.8 ± 1.3 (58)	3.406	0.01	0.11
Levels	13 ± 3 (49)	12 ± 3 (60)	−2.511	0.01	0.05
OP time (m)	254.5 ± 48 (46)	285 ± 57 (60)	2.859	0.004	0.077

^∗^indicates anterior releases.

**Table 2:** Characterization of the population under study before and after the implementation of the multimodal approach program to decrease transfusion needs in patients with scoliosis associated with neuromuscular syndromic and muscular dystrophies diseases (Group B).

Characteristic	Before MAP (B1) % (N)	After MAP (B2) % (N)	Pearson chi-squared (Mann–Whitney)	*p* value	OR (95% CI) (eta-squared)
Gender	50% (52)	58% (48)	0.698	0.406	1.4 [0.63; 0.57]
Weight (kg)	38 ± 16.5 (40)	40 ± 24.8 (45)	1.194	0.233	0.016
Age (years)	13.5 ± 5 (52)	13 ± 4 (48)	−0.097	0.923	<0.0001
Cobb angle (°)	79.5 ± 38.8 (52)	83.9 ± 30.15 (48)	0.821	0.412	0.006
Levels	16 ± 3 (52)	15 ± 2 (48)	−1.723	0.085	0.029
Adit. procedure^*∗*^	28.8% (15)	12.5% (6)	4.02	0.046	0.352 [0.12; 1.00]
Pre-op hb	13 ± 2.3 (48)	13.5 ± 1.4 (48)	1.008	0.313	0.01
OP time (m)	272.5 ± 60 (40)	300 ± 102 (47)	1.109	0.268	0.014

^∗^indicates anterior releases.

**Table 3:** Perioperative outcomes in patients with scoliosis associated with idiopathic deformities and congenital treated as idiopathic before and after multimodal approach program (MAP).

Characteristic	Before MAP (B1) % (N)	After MAP (B2) % (N)	Pearson chi-squared (Mann–Whitney)	*p* value	Eta-squared OR (95% CI)
Hg intra-op	9.8 ± 3 1.7 (49)	10.3 ± 1.8 (59)	1.488	0.137	0.02
Hg post-op day 1	9.7 ± 1.4 (48)	9.2 ± 1.6 (60)	−1.38	0.66	0.017
Hg low post-op	9.1 ± 1.5 (45)	8.75 ± 1.43 (60)	−1.344	0.179	0.017
Hct low intra-op	29.3 ± 3.5 (40)	30.1 ± 4.95 (53)	0.63	0.529	0.004
Hct post-op day 1	28.7 ± 4.4 (40)	27.3 ± 5.1 (53)	−0.572	0.567	0.003
Hct low pos-op	27.7 ± 3.8 (40)	25.5 ± 5.05 (53)	−1.153	0.123	0.025
Total losses	500 ± 450 (37)	460 ± 300 (59)	−1.519	0.129	0.02
Losses/vol (%)	13.1 ± 9 (32)	12.3 ± 11(56)	−0.013	0.99	<0.001
Losses/level	41.7 ± 31.4(37	39.29 ± 23.33 (59)	−0.61	0.542	0.004
Transf. rate (%) (n)	83.7% (49)	28.3% (60)	33.178	<0.001	0.077 [0.03; 0.198]
Transf. index (N/pt)	2 ± 1(49)	0 ± 1	−6.347	<0.001	0.37

**Table 4:** Perioperative outcomes in patients with scoliosis associated with scoliosis associated to neuromuscular, syndromic and muscular dystrophies before and after multimodal approach program (MAP).

Characteristic	Before MAP (B1) % (N)	After MAP (B2) % (N)	Pearson chi-squared (Mann–Whitney)	*p* value	Eta-squared OR (95% CI)
Hg intra-op	9 ± 2.35 (49)	9.65 ± 2.25(48)	1.488	0.137	0.023
Hg post-op day 1	9.65 ± 2.7 (48)	9.5 ± 1.4 (48)	1.488	0.137	0.023
Hg low post-op	8.5 ± 1.8 (48)	8.4 ± 2.08 (48)	1.386	0.166	0.02
Hct low intra-op	26.3 ± 6.4 (40)	28.4 ± 6 (39)	1.992	0.046	0.046
Hct post-op day 1	25 ± 4.75 (40)	24.6 ± 5.5 (39)	−1.691	0.091	0.032
Hct low pos-op	28.3 ± 7.3 (40)	27.6 ± 5.2 (39)	−0.433	0.665	0.002
Total losses	600 ± 500(43)	500 ± 550 (47)	−1.028	0.304	0.012
Losses/vol (%)	21.4 ± 13.3 (37)	16.6 ± 14.4 (45)	−2.232	0.026	0.06
Losses/level	43.3 ± 32.35(43)	37.5 ± 37.5	−1.176	0.240	0.015
Transf. rate (%) (n)	98.7% (52)	66% (47)	17.906	<0.001	0.038 [0.05; 0.30]
Transf. index (N/pt)	3 ± 3 (52)	1 ± 2	−5.373	0.009	0.29

**Table 5:** Regression coefficients of the standardized linear models.

Dependent variable	B expo (*p* value)	B EXPO diagnosis B (*p* value)	Model R2
*Z*(EBL (ml))	−0.433 (0.003)	0.216 (0.189)	0.209
*Z*(EBL/volemia (%))	−0.257 (0.084)	0.539 (0.001)	0.241
*Z*(EBL/levels fused (ml))	−0.416 (0.005)	0.330 (0.066)	0.171

**Table 6:** Logistic regression for variables which affect the occurrence of a transfusion.

		B	SE	Wald	df	Sig.	Exp (B)
Step 1^a^	ZHgp	−7.23	0.259	7.776	1	0.005	0.485
	ZCobbM	1.038	0.308	11.326	1	0.001	2.824
	DIAG_BI	1.480	0.467	10.037	1	0.002	4.392
	Anest	−0.999	0.485	4.237	1	0.040	0.368
	AcTrax	−2.332	0.507	21.112	1	0.000	0.097
	Constant	1.870	0.437	18.307	1	0.000	6.487

^a^Variable(s) entered on step 1: ZHgp, ZCobbM, DIAG_BI, Anest, and AcTrax. Calculations were performed with the standardized value for continuous variables. Categorical variables were defined as dummies with 1 for “yes” and 0 for “no.” The advantages of using standardized variables are related to the better understanding of relative ODDS in the presence of continuous and categorical variables as predictors. To use this regression as a predictive value, the following should be considered: the “Cobb” variable has a normal distribution with mean = 74.716 and SD = 22.621; the variable “HgP” is also normally distributed with mean = 13.192 and SD = 1.4329. To use the provided coefficients, the users should follow the formula: logit = 1.870–0.723 [(HgP-13.192)/1.4329] + 1.038[(Cobb-74.716)/22.621] − 0.99(remifentanil) − 2.332(tranexamic acid) + 1.480 (neuromuscular diagnosis) and entering the HgP value and Cobb angle of their patient. To transform the logit into a more familiar concept of probability (P), the formula to be used is *p* = EXP (logit)/[EXP (logit) + 1].

## Data Availability

The data presented within this manuscript are available upon request from the first author (pfpcm09@gmail.com).
